# Description of control measures, attitudes, and behaviours at a scientific conference with a confirmed COVID-19 case but no reported onward transmission, November 2021 England

**DOI:** 10.1016/j.puhip.2024.100521

**Published:** 2024-06-20

**Authors:** Katie Wrenn, Paula Bianca Blomquist, Petra Manley, Jin-Min Yuan, Ellie Gilham, Hannah Higgins, Andrew Curran, Yiqun Chen

**Affiliations:** a Field Services, UK Health Security Agency, London, UK; b Science Division, Health and Safety Executive, Buxton, UK

**Keywords:** COVID-OUT, SARS-CoV-2, COVID-19, Outbreak, Scientific conference

## Abstract

**Background:**

COVID-19 (coronavirus disease 2019) outbreaks in workplace settings have been investigated to understand how transmission occurred. However, there is limited data looking at COVID-19 transmission in conference settings in England, particularly where an outbreak did not occur. The aim of this work was to investigate COVID-19 infection risk factors and control measures at a large conference, with a known case but no reported onward transmission to inform prevention of future outbreaks of respiratory infections in conferences and similar settings.

**Methods:**

This cross-sectional study was part of a wider COVID-19 Outbreak Investigation to Understand Transmission (COVID-OUT) study. A two-day in-person conference on SARS-CoV-2 transmission and environment was held at a university conference centre on 17–November 18, 2021, in England, with about 100 delegates. A questionnaire survey was conducted among 50 conference attendees to identify any confirmed cases and understand transmission, history of COVID-19 symptoms, testing and vaccination.

**Results:**

One person met the definition of a confirmed case at the conference. This case was most likely infectious when attending the conference, however there were no known secondary cases. All respondents reported receiving at least two doses of a COVID-19 vaccine before the conference and an increased frequency of handwashing/sanitising hands during the study period in comparison to before the pandemic. Prior to the conference, a COVID-19 risk assessment including a review of the ventilation at the site was completed. All attendees were advised to take an LFD test before travelling to the conference, wear face coverings, and maintain 1-m distance during the conference.

**Conclusion:**

A multipronged approach, encouraging attendee behaviours (regular hand washing, mask wearing, being vaccinated against COVID-19) and introducing control measures at the conference site (ventilation, sufficient spacing capacity, combined with prior knowledge of COVID-19 transmission, were effective in limiting the spread of COVID-19 in this setting.

## 1 Introduction

The coronavirus disease 2019 pandemic (COVID-19) saw control measures introduced globally to prevent the spread of the severe acute respiratory syndrome coronavirus 2 (SARS-CoV-2). The United Kingdom (UK) implemented its first national lockdown in March 2020, followed by a range of restrictions and guidance specific to locations [1]. Outbreaks were observed throughout the pandemic in a variety of settings, such as, prisons [2], workplaces [3] and care homes [4]. Understanding transmission in all settings where outbreaks have occurred and those where widespread transmission was not seen [5] provides useful insight in preventing future COVID-19 outbreaks.

A two-day in-person scientific conference on SARS-CoV-2 transmission and environment was held at a university conference centre on 17–November 18, 2021, in England, with about 100 delegates from different parts of the country. The conference involved meetings for all attendees in the plenary room, workshops in breakout rooms, a knowledge café and interactive science fair in large rooms, as well as two indoor lunches and a conference dinner. Three days after the conference, an attendee was diagnosed with COVID-19 and self-reported this to the conference organiser. An email was sent out by the conference organiser to all delegates on November 22, 2021 to advise them to follow the government guidance and instructions from NHS Test and Trace if they were contacted. As part of the COVID-19 Outbreak investigation to Understand Transmission (COVID-OUT) study, a questionnaire survey was conducted among the conference attendees, including the case, to investigate whether there were any further new cases of COVID-19 amongst the attendees, or any evidence of COVID-19 spread during the conference and to understand the context that affected transmission risk [6].

## 2 Methods

This cross-sectional study was part of the wider COVID-OUT study, which involved investigations of SARS-CoV-2 outbreaks in a range of workplace settings, using a pre-designed study protocol to identify outbreaks and their causes, including an in-depth questionnaire survey tool. The COVID-OUT study questionnaire was adapted to collect data for this scientific conference [6]. The voluntary online questionnaire was sent out to all conference attendees, including the conference support staff, two weeks after the conference. The questionnaire took approximately 30 min to complete and collected data on behaviours and whereabouts relevant to COVID-19 transmission as well as data on history of COVID-19 symptoms, testing and vaccination.

The questions referred to two time periods. The first covering anytime from February 2020 to the survey completion date, the second asking about the conference period (covering 2 days before the conference and 10 days after).

We described questionnaire uptake, patterns of self-reported lateral flow device (LFD) and polymerase chain reaction (PCR) testing, the number and proportion of individuals who reported COVID-19 compatible symptoms and those testing positive for COVID-19, over the conference period. We then used questionnaire data on social interactions, workplace settings, and attendee vaccination history, as well as details of COVID-19 control measures provided by the conference organisers to understand the overall level of transmission risk at the event.

The following definitions were used for this analysis.

- A confirmed case at the conference: an attendee reporting a positive PCR test from between 48 h prior to the conference or up to the 10 days after the conference (15–29 November).
- A suspected case: an attendee with no positive PCR results but presented during the study period with (i) a self-reported positive LFD test or (ii) no self-reported LFD test but symptoms consistent with COVID-19, defined as (a) acute onset of fever (>37.8 °C) and new continuous cough or (b) acute onset of any three or more symptoms of fever (>37.8 °C), cough, shortness of breath, loss of taste or smell, runny nose, fatigue, sore throat, muscle or body aches, headache, nausea or vomiting, and/or diarrhoea.

Ethical approval for the COVID-OUT study was provided by the NHS North East Research Ethics Committee (Reference 20/NE/0282). Participation in the COVID-OUT study was voluntary and was based on individuals’ informed consent. Attendees who gave their consent and took part in the survey were offered a £20 gift card as a compensation for their time spent to complete the questionnaire. Six COVID-OUT study researchers attended the conference. They could take part in the questionnaire survey but would not receive the £20 gift card. Any identifiable data from the questionnaire were kept confidential and were handled only by the small data management team who was not involved in the conference.

## 3 Results

### 3.1 Questionnaire uptake and testing

In total, 50 (42 %) of the 118 conference attendees, including 107 delegates and 11 support staff members from the catering, venue and audio visual teams, responded to the online questionnaire survey, with a mean age of 43.8 (range 22–68) (Table 1). In the 48 h before attending conference, LFD tests were undertaken by 84 % (37/44) of participants who reported attending the conference on day 1, and all received negative results. Three participants additionally took a PCR test, and all received negative results.

**Table 1:** Overall counts of conference attendees and questionnaire respondents.

	Conference attendees (n)	Questionnaire respondents (n (%))
**Overall count**	118	50 (42 %)
**Attending on both conference days**	–	40 (80 %)
**Attending only on day one (November 17, 2021)**	–	4 (8 %)
**Attending only on day two (November 18, 2021)**	–	6 (12 %)

LFDs were undertaken by 50 % (3/6) of new attendees before attending only on day 2. An additional 7 attendees from day 1 repeated their test before day 2. All results were negative.

**Fig. 1:**
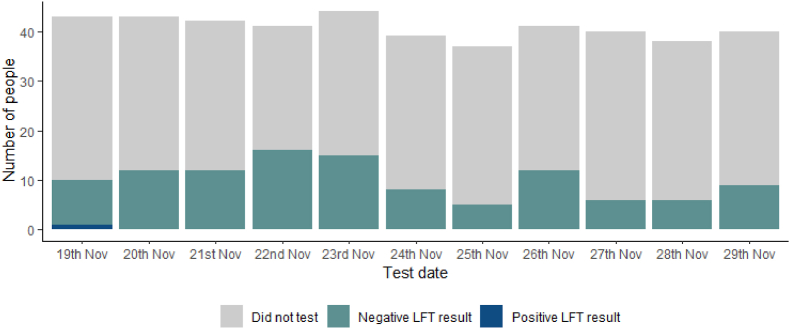
Patterns of testing among respondents on the days immediately after the conference: 19th – November 29, 2022.

After the conference, between 19 and November 29, 2021, 42 respondents (84 %) reported taking at least one LFD test, however these tests were spread over the entire period (Fig. 1.). The largest proportion of LFD tests were taken on 22nd November (32 %), when an email was sent out to all attendees about the positive case who had attended the conference; 41 % of respondents reported performing more than one test over the conference period.

### 3.2 Confirmed and suspected COVID-19 cases

One person met the definition of a confirmed case at the conference, with symptom onset on day 2 followed by a positive LFD test the day following the conference on 19th November, and then a confirmatory PCR result on 20th November. This case had taken two previous LFDs on 15th November and 17th November, both of which came back negative. The case travelled to the conference by train, attended all conference activities on both days, stayed overnight at a hotel accommodation after the day one of the conference. The case started to feel unwell in the afternoon of the second day of the conference and developed a cough on the way home. This case notified the conference organiser of their positive test result after the conference.

No respondents met the definition of a suspected case, with no COVID-19 symptoms reported in those that did not report taking an LFD test.

14 respondents did report possible COVID-19 symptoms during the study period (15–29 November). However, all these cases undertook at least one LFD test during this time and received negative results. Runny nose was the most common symptom reported, followed by sore throat, headache, fatigue, and muscle/body aches.

### 3.3 Survey respondent behaviours during the conference period (Table 2)

The most common mode of transport, used as part of the respondents’ journeys to and from the conference was train or tram (82 %) followed by private car (24 %).

Respondents reported an increased frequency of handwashing/sanitising hands at work between 15 and 29 November in comparison to before the pandemic. 20 % of respondents reporting washing their hands 11 to 20 times a day during the conference period and only 6 % reporting this before the pandemic.

**Table 2:** Count of respondent behaviours over the conference period (n = 50).

Respondent behaviours between 15th to 19th November	n (%)
Stayed overnight at hotel or B&B	31 (62 %)
Downloaded the COVID-19 app and enabled Bluetooth	20 (40 %)
Were notified by the COVID-19 app of a close contact (All were after the conference and none during the conference.)	7/20 (35 %)
Worked in a non-home setting	43 (86 %)
Wore a face covering “nearly all the time” in an indoor public space (not including work and the conference)	23 (46 %)

### 3.4 Respondent daily close contacts

Between 15 and 29 November, the highest proportion (88 %) of respondents reported an average of 1–5 close contacts a day at the place that they live. This was compared to 54 % at the place for social activities, 46 % for essential activities and 42 % at the workplace. A close contact is defined as spending >15 min within 2 m of someone. The most frequent settings, where the respondents reported spending on average more than 1 h at least once a day in the same room or confined space with someone who was not in their household, was at the workplace (24 %). This was compared to 22 % at the place they live, 10 % when commuting for work and another 10 % during social activities. Eight respondents reported close contacts with a positive COVID-19 case and six of them stated that the case was someone they worked with.

### 3.5 Respondent workplace setting

42 % of respondents reported working in academia and 36 % in government. At work, 92 % of respondents reported not having much direct physical or close contact with co-workers or members of the public. Of the 48 respondents who worked mainly indoors, 85 % reported a good supply of fresh air in the workplace with five reporting “I don't know” and two reporting “No”. 94 % (47/50) of respondents had received training about preventing COVID-19 in the workplace. 96 % (48/50) of respondents anticipated no changes to their pay if they were required to self-isolate, but when asked about worries with reduced income due to self-isolation, two of the 48 respondents were slightly worried, and a further two were very worried.

### 3.6 Respondent past infection and vaccination history

All respondents, including the case reported receiving at least two doses of a COVID-19 vaccine. With the case receiving doses in March and May 2021. 16 people reported also receiving a third booster dose. Four respondents reported having a previous COVID-19 infection confirmed by PCR testing.

### 3.7 COVID-19 control measures at the conference

A COVID-19 risk assessment was completed prior to the conference start date, which concluded the conference centre was suitable to host the event whilst posing minimal risk of COVID-19 transmission. Ventilation at the site was reviewed by internationally recognised airborne transmission and ventilation experts, who ensured ventilation met the UK standard for the number of people expected to attend the conference. To minimise contact with those outside of the conference, there was a dedicated floor and lift for attendees. One metre social distancing was implemented during the conference, as the size of the venue allowed for sufficient spacing capacity. Seating and activities were also organised to ensure this distance was kept, for example, a vacant seat between attendees in meetings. Portable HEPA (High-Efficiency Particulate Air) filter air purifiers and CO_2_ monitors were observed in various positions. Hand sanitiser points were located throughout the venue to encourage regular hand sanitising. Attendees were sent an email prior to the conference encouraging them to take an LFD test before travelling to the conference and told not to attend if receiving a positive result or showing any COVID-19 symptoms. Advice on wearing face coverings at all times (except when eating, drinking or presenting) and maintaining a 1 m distance during the conference period was also issued.

Of questionnaire respondents, 92 % (46/50) received and read an email about COVID-19 preventative control measures prior to the start of the conference, with three (6 %) not receiving this email and one individual not responding to this question. 94 % of respondents rated the conferences COVID-19 preventative control measures as excellent or above average.

## 4 Discussion

In a scientific conference hosting 118 people, this study identified only one positive COVID-19 case who might have been infectious during the conference, and no known secondary cases. This may be an underestimate as not all attendees engaged with the study and not all respondents tested for SARS-CoV-2. Out of those who did test, many tested only once after the conference and the majority did so 0–4 days following the suspected exposure [7]. However, the study questionnaire survey was started two weeks after the conference which should have been sufficient to capture data on relevant COVID-19 tests or symptoms. Additionally, while LFD tests have been shown as an effective method of identifying asymptomatic SARS-CoV-2 infections that would have not otherwise been detected [8], they may not pick up infection due to lower infectivity or user error [9].

At the time that the conference was held, most legal limits on social contact had been removed in England [10]. The positivity rate in the local area was 1.4 % and around 86 % of individuals in England reported to have received two doses of a COVID-19 vaccine [11,12]. The Delta variant (B.1.617.2) was the most dominant strain in the England at the time of the conference, with the case occurring at the start of the biggest wave of infections seen in the country over the course of the pandemic. It is important to note that in different context with a higher infection rate, alternative circulating variant and/or tighter restrictions, transmission risk would have been different. However, a similar outcome was observed at a conference held in Geneva with a higher local incident rate [13] and at a conference held in Florida hosting 1617 people, when cases with Omicron were on the rise. In Florida, they adapted similar safety measures to those in this study and did not see increased COVID-19 positivity amongst in-person attendees in comparison to virtual attendees [14].

Regular testing, hand hygiene, mask wearing, and social distancing have all been shown effective in reducing the transmission of COVID-19 [15]. The conference facilitated and encouraged all these control measures. Evidence of increased frequency in hand washing during the conference period in comparison to frequency before COVID-19, suggests that hand sanitiser points were utilised. The conference also ensured effective ventilation, which has been shown as a strategy for controlling COVID-19 transmission [16]. The conference was set up to minimise the risk of environmental factors on the spread of COVID-19 in this setting. With most respondents ranking the COVID-19 control measures at the conference as high and reporting performing an LFD test 48 h before the conference, it suggests control measures were well communicated and implemented.

Knowledge, attitude, and behaviour can influence COVID-19 transmission [17]. Therefore, training received by respondents on preventing COVID-19 transmission at work is likely to have encouraged individual behaviours that tend to reduce the likelihood of transmission. Similarly, compliance to public health regulations is increased when individuals have assurance about their livelihood [18]. Nearly all respondents in this study were not worried about reduced income if they needed to self-isolate, hence were more likely to agree to performing LFD tests during the conference.

The highest proportion of respondents reported working in academia, which when compared to the workplace setting of essential workers, puts this group at much less risk of infection [19]. It is therefore important to consider environments attended outside of the conference when understanding risk of transmission at this event.

This was a small-scale conference, however, an indoor convention in New York City with 53,000 attendees from 52 U S. jurisdictions and 30 foreign countries showed similar results. The study showed vaccination requirements, enforced mask use, avoidance of unmasked indoor settings, and a venue with HEPA filtration were likely to account for few event-associated cases [20].

All responding attendees to the conference, including the case had received at least two doses of a COVID-19 vaccine. In a setting with unvaccinated attendees results might have been different. This was seen at the New York City convention where fewer infections were seen among those who had received their vaccine booster dose [21].

This study suggests that several measures may have collectively limited the spread of COVID-19 in this setting, as also seen in a study looking into safety measures at a conference in Mexico. Here a multipronged approach, of environmental and behavioural factor was thought to be most beneficial to mitigate risk of SARS-CoV-2 infection [22].

To conclude, this study has shown that at a time of rising infections in England, introducing multiple control measures at a conference site (ventilation, sufficient spacing capacity, prior advice on preventing transmission), in addition specific behaviours performed by attendees (regular hand washing, mask wearing, being vaccinated against COVID-19) limited transmission of COVID-19 in this setting. Though the study doesn't show which factors were most influential in reducing transmission, it does indicate that when combined these measures can be a useful tool. Demographics and workplace setting in this group meant they had prior knowledge and training around COVID-19 transmission and were less worried about self-isolation due to COVID-19, which likely influenced their behaviours and will have been a key factor in why the control measures were effective. This multipronged approach, encouraging attendee behaviours and introducing control measures at the conference site, could be used as a model when looking to limit the spread of COVID-19 at a conference or similar settings in the future.

## Author statements

YC and ADC conceived the study. EG and HH managed the online questionnaire and assisted in data curation. J-MY and KW analysed the survey results supported by PB, YC and PM. KW and YC wrote the manuscript, supported by PB, PM and ADC. All authors provided input into the final manuscript.

## Ethical approval

Ethical approval for the COVID-OUT study was provided by the NHS North East Research Ethics Committee (Reference 20/NE/0282).

## Competing interests

The authors declared no conflicts of interest.

## Funding statement

This work was funded by the PROTECT (Partnership for Research in Occupational, Transport and Environmental COVID Transmission) COVID-19 National Core Study on Transmission and Environment and managed by the 10.13039/501100000869Health and Safety Executive on behalf of 10.13039/100013986HM Government.

## Copyright

© Crown copyright (2023) Health and Safety Executive. This is an open access article distributed under the terms of the Open Government Licence v3.0, which permits re-use, distribution, reproduction, and adaptation, provided the original work is properly cited.

## Disclosure statement

The contents of this paper, including any opinions and/or conclusions expressed, are those of the authors alone and do not necessarily reflect Health and Safety Executive or UK Health Security Agency policy.

## Declaration of competing interest

The authors declare that they have no known competing financial interests or personal relationships that could have appeared to influence the work reported in this paper.
